# Experimental elevated temperature affects bumblebee foraging and flight speed

**DOI:** 10.1098/rspb.2024.1598

**Published:** 2024-10-30

**Authors:** Maxence Gérard, Erika Gardelin, Philipp Lehmann, Kevin T. Roberts, Guadalupe Sepúlveda-Rodríguez, Clara Sisquella, Emily Baird

**Affiliations:** ^1^ Department of Zoology, INSECT Lab, Division of Functional Morphology, Stockholm University, Svante Arrhenius väg 18b, 11418 Stockholm, Sweden; ^2^ Laboratory of Zoology, Research Institute for Biosciences, University of Mons, Place du parc 20, 7000 Mons, Belgium; ^3^ Department of Animal Physiology, Zoological Institute and Museum, University of Greifswald, 17489 Greifswald, Germany

**Keywords:** *Bombus terrestris*, climate change, flight, global warming, insect, pollination

## Abstract

Global warming threatens wild bees and their interaction with plants. While earlier studies have highlighted the negative effects of elevated temperatures on bee–plant interactions, we still lack knowledge about how they impact the foraging behaviours that are central to bee pollination activities. To address this knowledge gap, we investigated how ambient temperature affected the foraging behaviours of the bumblebee *Bombus terrestris*. We allowed the bumblebees to forage freely on artificial flowers in two climate-controlled rooms set at 24°C and 32°C. The colonies were alternated between the two temperatures every week. We recorded the flower visitation rate, flight speed, total foraging time and number of foraging trips. In addition, we measured flight metabolic rate across a range of temperatures to assess its potential as an underlying mechanism. In comparison to 24°C, at 32°C, flower visitation time decreased while flower visitation rate and flight speed increased. This is consistent with the reduction in flight metabolic rate recorded between these temperatures. At 32°C, the number of trips made by each worker decreased, suggesting that, despite the reduced energetic cost, flight in elevated temperatures may be stressful. Our results suggest that elevated temperatures affect bumblebee foraging behaviour and that this would likely disrupt plant–insect interactions.

## 1. Introduction

Insect pollinators are a crucial functional group for both wild plant communities and agricultural productivity [1] but they are globally declining in both abundance and diversity [2–4]. Climate change represents one of the greatest threats to insect pollinators and the ecosystem services they provide [3]. Exposure to elevated temperatures can affect insect development, morphology, metabolism and behaviour [5–9], or any trait that is sensitive to temperature [10]. More specifically, temperature can determine if or how a given behaviour will be expressed [11]. Elevated temperatures can impact insect behaviour both directly through the kinetic effects on metabolic rate [6], and indirectly through behavioural decisions to minimize exposure [11], such as minimizing the time of a foraging trip, but maximizing the income during this trip [12].

The impact of temperature on insect foraging behaviour—particularly with respect to pollination—is one important but hitherto overlooked aspect of global warming studies. Moreover, the effect of temperature on the metabolic rate of insect pollinators is poorly understood (but see [13]), despite being a critical driver of foraging behaviour. As ambient temperature increases, insect metabolic rates typically increase up to a critical upper limit. The following decrease is associated with increasing stress and, ultimately systemic cell death [5]. By changing foraging behaviour, elevated temperatures could thus impact how insect pollinators interact with plants [14,15]. For example, colonies of the bumblebee *Bombus terrestris* that were exposed to elevated temperatures (33℃) during worker development had fewer foragers and the time each individual spent on flowers was reduced in comparison to colonies reared under an optimal temperature (27°C) [16]. Elevated ambient temperatures (35℃) also reduced the time that *B. impatiens* workers spent foraging and the number of successful foraging trips [17]. While the results of previous studies imply that global warming will disrupt plant–pollinator interactions [15,18], our understanding of the underlying mechanisms is poor.

To address this knowledge gap, we characterize the effect of elevated ambient temperatures on the foraging behaviour of the bumblebee *B. terrestris* and explore whether these changes can be explained by temperature-induced changes in flight metabolic rate. We recorded the foraging trips of workers at two different ambient temperatures—24 ± 1℃ and 32 ± 1℃—while maintaining the colonies at an optimal temperature of 26°C [19,20] to ensure that only individuals outside of the colony were affected by the experimental treatments. The optimal temperature condition reflects an ambient temperature that *B. terrestris* would experience when foraging in spring and summer in temperate regions and lies within the optimal temperature range for distance and duration of flight [21]. The elevated temperature condition lies at the higher end of the ambient temperature range for this species and has been shown to affect cognition [15] and worker fanning activity [20,22]. We hypothesize that elevated ambient temperatures affect bumblebee foraging behaviour and that this is related to temperature-induced changes in the flight metabolic rate [23]. We also hypothesize that, in the elevated temperature, workers will avoid overheating by changing their foraging decisions: we predict that at high temperatures they will perform fewer foraging trips and decrease the overall foraging time (i.e. the time spent foraging outside of the colony).

## 2. Material and methods

### (a) Bumblebee maintenance

Commercial colonies of the bumblebee *Bombus terrestris* (Koppert, Berkel en Rodenrijs, The Netherlands) were used in this study. Upon arrival, 100 workers from each colony were randomly selected and colour-coded numbered plates (LPs Biodling, Säffle, Sweden) were fixed to their thorax for individual identification (ID). The remaining unmarked individuals (except for the queen) were removed to standardize the colony size. Individuals that emerged during the course of the experiment were also tagged. The colonies were placed in dark incubators kept at 26℃ and 60% relative humidity for optimal developmental conditions [20,22]. The colonies had ad libitum access to sucrose solution (Koppert Natupol smart sucrose solution) and were provided with one pollen candy (consisting of a mixture of fresh-frozen organic pollen and 1 : 1 water : sugar solution from Naturprodukter, Rawpowder Bipollen) every week.

### (b) Foraging experiment set-up

**Figure 1.**
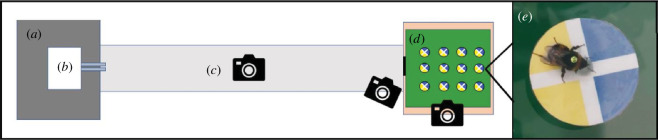
Experimental set-up and camera placement from the top with incubator (*a*), hive (*b*), flight tunnel (*c*) and foraging arena (*d*). A tagged bumblebee foraging on an artificial flower in the foraging arena is shown in (*e*).

Identical experimental set-ups consisting of an incubator, a flight tunnel and a foraging arena were assembled in two climate-controlled rooms with the ambient temperature set to 24 ± 1°C or 32 ± 1°C. While the flight tunnel and the foraging arena were exposed to these ambient temperatures, the incubators in both rooms were regulated at 26°C. Both rooms had a relative humidity of approximately 30% and had a 12 : 12 controlled light–dark cycle with light from 07.00 to 19.00. The incubators containing the colonies were connected via two plastic tubes (15 cm long, 15 mm diameter) to the flight tunnel (200 cm × 30 cm × 30 cm) that was, in turn, connected via a gate to a foraging arena (60 cm × 60 cm × 40 cm; figure 1). The walls of both the flight tunnel and the foraging arena were lined with patterned paper to provide naturalistic visual feedback (i.e. a black-and-white ‘dead leaves’ pattern on the left and right walls of the tunnel [24,25]). The foraging arena floor was covered with green plexiglass to provide contrast against the artificial flowers. Three cameras were placed in each room to record activity (figure 1): one recorded the flights to and from the foraging arena, one recorded the identity tags of bees entering the foraging arena and one recorded the activity in the foraging arena.

Artificial flowers were used in the experiment to standardize the sugar reward and to avoid variability in flower traits that could affect visitation. These flowers comprised 0.2 ml Eppendorf tubes filled with 1 : 1 water : sugar solution (hereafter referred to as sucrose solution) placed at the centre of laminated paper cut into circles (45 mm in diameter) with half-blue half-yellow colouration and white crosses serving as nectar guides (figure 1) to promote faster localization and successful visits to the flowers [26]. The flowers were placed into 12 magnetic tubes distributed in 3 × 4 rows at 10 cm spacing and fixed to magnets on the arena floor to facilitate replacement and cleaning.

### (c) Foraging experiment design

The day after being marked with ID tags, the colonies were provided access from the incubator to the experimental set-up. During this day, bees were provided with sucrose solution and ground pollen at a sugar–water feeder as well as six artificial flowers used in the experiments. The feeder was initially placed near the hive entrance and, once several bees were feeding from it, moved successively along the tunnel. During this phase, 12 artificial flowers filled with sucrose solution and dusted with pollen were also placed inside the foraging arena. The following day, the feeder and the six flowers in the flight tunnel were removed, leaving only the 12 flowers in the foraging arena and a tray with ground pollen. The number of bees foraging during the experiment in the arena was not restricted. When the first workers learned to feed from the flowers, all pollen was removed, and the training phase was complete.

**Figure 2.**
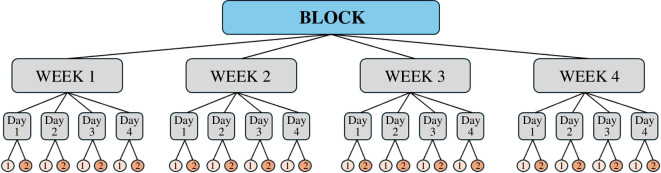
Chart of how each experimental block (consisting of two colonies) was divided into weeks, days and trials. The trials are the subdivision within a day, corresponding to the experiment run each morning (1) and afternoon (2).

The experiments were divided into three blocks with two colonies per block, resulting in a total of six colonies. Each experimental block lasted 4 weeks and consisted of four consecutive days of recording per week (figure 2). Two 30 min recordings (defined here as trials) per treatment were conducted per experimental day (09.00−09.30 and 13.00−13.30; figure 2) to control for potential differences in foraging activity across the day [27]. This resulted in 32 trials per colony or 64 trials per experimental block (as two colonies were tested during each block), totalling 192 trials. When the bees were not participating in these trials, they always had access to sugar solution ad libitum in the arena. This was ensured by placing a sugar−water feeder in the arena during non-working hours or lunch break, or because other experiments requiring the flowers to be filled were conducted. Moreover, we have never observed that any bees emptied a flower during the 30 min trial, which avoids making a flower ‘unrewarding’. The colonies were categorized into three different temperature treatment sequences (with each temperature in the sequence representing one week; see table 1 for a description week by week of each temperature treatment and the corresponding colony): (a) 24°C−32°C−24°C−32°C, (b) 32°C−24°C−32°C−24°C and as a control (c) 24°C−24°C−24°C−24°C (alternated between rooms).

**Table 1.** The experimental blocks and sequences with corresponding temperature treatment per week and colonies.

colony ID	experimental block	sequence	week 1	week 2	week 3	week 4
A	1	1	24°C	32°C	24°C	32°C
B	1	2	32°C	24°C	32°C	24°C
C	2	2	32°C	24°C	32°C	24°C
D	2	1	24°C	32°C	24°C	32°C
E	3	3	24°C	24°C	24°C	24°C
F	3	3	24°C	24°C	24°C	24°C

### (d) Foraging parameters

Five foraging parameters were measured at the individual level: visitation rate, visitation time, flight speed, foraging time and the number of foraging trips. Visitation rate was defined as the number of flowers visited by a bumblebee during 1 min of foraging activity, leading to a rate of flowers visited per minute. Visitation rate is a component of the pollination efficiency and is usually positively correlated to this variable [28]. In terms of colony development, a higher visitation rate can also increase the quantity of resources brought back to the colony per unit of time [29]. Yet, Ne’eman *et al.* [28] highlight that it cannot explain pollination efficiency alone. Visitation time was defined as the time (taken using a stopwatch) between the first and last contact with the same flower. Flight speed was measured on foragers from colonies A, B, C and D flying in the two experimental temperatures. Videos from the flight tunnel were digitized in MATLAB (The Mathworks Inc.) with the app DLTdv8a (v. 8.2.10) and calibrated into real-world coordinates using known points in the tunnel and known distance markers. Flight speed was calculated as the sum of the two-dimensional distance travelled over 50 cm in the centre of the tunnel divided by time and using only the workers flying from the colony to the arena (as workers leaving the arena have experienced different times/temperatures outside of the colony). Foraging time was defined as time spent in the foraging arena, from the moment a bumblebee entered it to the time it left or had been inactive in the arena for 3 min. Everything equal, increasing flight speed allows to increase the efficiency of a foraging flight, by decreasing the foraging time, and thus bringing back more resources per unit of time. Data on foraging time and visitation rate were only taken from the first 10 min of each trial due to the large number of foraging bumblebees. Number of foraging trips was defined as the number of foraging trips made by an individual in one single trial. Increasing the number of foraging trips increases the number of resources brought back to the colony. Flowers were refilled with sugar water during each trial if they had been nearly emptied and before the start of each new trial. All flowers were cleaned with 70% ethanol between trials to minimize the effect of potential scent cues left by bumblebees. Two parameters were measured at the treatment level (i.e. the sum of these parameters over the whole experiment, per temperature): the total number of workers foraging and the total number of foraging trips. To make the values comparable, we only included these parameters from colonies A to D, as they experienced the same number of trials per temperature. Data taken by observers were confirmed using the video recordings to control for potential errors when noting IDs and foraging activity.

### (e) Flight metabolic rate measurements

Flow-through respirometry was used to measure the metabolic rate of flying bumblebees. To describe how flight metabolic rate varies across the temperatures tested during the foraging experiments (i.e. 24°C and 32°C), we recorded the flight metabolic rate of workers at four temperatures: 24°C (*n* = 10), 28°C (*n* = 11), 32°C (*n* = 12) and 36°C (*n* = 9) (42 workers in total with 20 and 22 individuals from two different colonies). Bumblebees had access to sucrose solution (Koppert Natupol smart sucrose solution) inside the colony and the colonies were supplemented with pollen for the brood every 3 days for the duration of the experiment. Our preliminary experiments showed that bees did not sustain flight for longer than a few seconds at temperatures above 36°C. We, therefore, included temperatures up to and including 36°C to explore how the metabolic rate is affected at the upper limits of the conditions under which flight can be sustained in the flight chamber. Bees exiting the colony were placed in individual tubes for an acclimation period of 30 min before metabolic rate measurements were conducted. Then, individual bees were placed in a custom-made 250 ml cylindrical flight chamber placed in an incubator (Panasonic MIR-154-PE) at different temperatures. Each bumblebee was only exposed to one temperature to avoid the effects of cumulative heat stress on flight metabolism. In current air was scrubbed of CO_2_ and H_2_O using Ascarite (Arthur H. Thomas Company, A0403541, Philadelphia, PA, USA) and Drierite (CaSO_4_, Sigma Aldrich, 7778-18-9, St Louis, MO, USA) columns and CO_2_-free air flowing at a rate of 700 ml min^−1^ was pumped through the respirometry chamber. Excurrent air went into an Ascarite column to remove H_2_O and CO_2_ content was measured using a CA-10 CO_2_ analyser (Sable Systems International, Las Vegas, USA). A UV-light (Dibotech, 64 210 IK92455) was used to stimulate flight and only flight bouts sustained for more than 60 s were used for the analysis, as this was significantly longer than flights recorded in the flight tunnel. Bumblebee flights from the colony to the foraging arena were approximately between 4 and 15 s long. Bumblebees were weighed and only the mass-corrected metabolic rates were used.

### (f) Statistics

All statistical analyses were performed using packages (highlighted in italics below) in R [30]. The five different foraging parameters were used as response variables for each separate model and random effects were the experimental block, the day nested in the experimental block, colony ID and individual ID nested in the colony ID. Temperature, sequence and their interaction were used as fixed effects in each full model. When the sequence did not have a significant impact on the response variable, we only used data from four colonies (A to D), to balance our dataset and have the same number of colonies experiencing the two temperature treatments in the analysis. The total number of foraging trips and workers performing foraging trips were only assessed through visual plots, as the total count resulted in one value per temperature treatment. After testing for other possible model structures and to account for the nonlinear relationship between metabolic rate and ambient temperature, a polynomial model was used to test whether temperature impacted bumblebee flight metabolic rate. In this model, temperature, colony ID and their interaction, as well as body mass were used as fixed factors.

All models about the foraging behaviour were tested for assumptions using *DHARMa* [31]. In the cases where assumptions were not met with log transformation, a generalized linear mixed model (GLMM) with Gamma distribution was built with *lme4* [32], with the exception of the model of number of foraging trips (i.e. count data), which was analysed with a GLMM with Poisson distribution. The final models were the models with the lowest AICc, tested using *AICcmodavg* [33]. In cases where the next best models based on ΔAICc did not significantly differ from the best model, the simplest model was used. ANOVA on the models was done using *car* [34], *post hoc* tests (and Bonferroni adjustment) using *emmeans* [35] and *multcomp* [36]. All data and R code related to this article can be found on Dryad [37].

## 3. Results

### (a) Visitation rate

**Figure 3.**
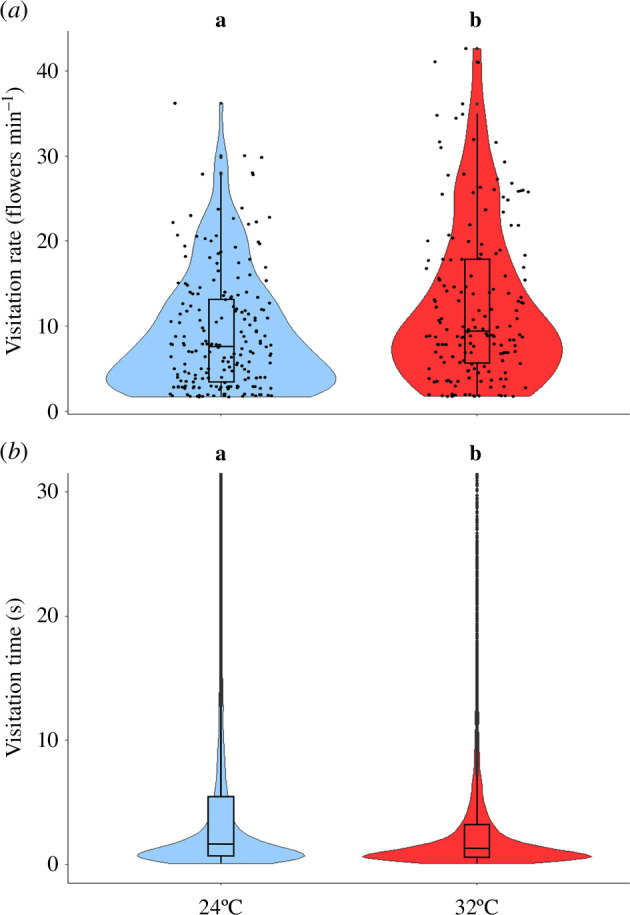
Visitation rate (*a*; *β* = 0.61, s.e. = 0.166, *t*-value = 3.687, *p* < 0.001) and visitation time (*b*; *β* = 0.09, s.e. = 0.006, *t*-value = 14.202, *p* < 0.001), results based on six colonies (A to F). Each dot in (*a*) represents one individual. Due to the large number of data points in (*b*), visitation times longer than 30 s are not shown (although they were included in the analyses) and data points have been omitted for clarity. Letters above the boxplots indicate significant differences when the letters are different. The central line within the boxplots represents the median. The upper and the lower lines represent the lower (Q1) and the upper (Q3) quartiles, respectively. The whiskers extend from Q1 and Q3 up to 1.5 times the interquartile range.

Visitation rate was recorded for 372 flights from 85 different individuals. The full model best explains the respective log visitation rate (next best model ΔAIC 3.33; electronic supplementary material, table S1). Visitation rate at 32°C was significantly higher than at 24°C (13 ± 7 flower min^−1^ versus 9 ± 8 flower min^−1^, *β* = 0.61, s.e. = 0.166, *t*-value = 3.687, *p* < 0.001; figure 3*a*), and the effect of sequence was not significant (*β* = 0.21, s.e. = 0.206, *t*-value = 1.014, *p* = 0.417).

### (b) Visitation time

We recorded 23 158 visitation times from 189 different individuals. Visitation time was best explained by a model that included temperature treatment, sequence and their interaction as fixed factors and experimental block and individual ID set as random factors (next best model ΔAIC 1.85; electronic supplementary material, table S2). A gamma distribution was used for the residuals. Visitation times at 32°C were significantly shorter than at 24°C (4.4 ± 9.4 s versus 7.0 ± 13.0 s; *β* = 0.09, s.e. = 0.006, *t*-value = 14.202, *p* < 0.001; figure 3*b*). While there was no significant impact of the sequence alone (*β* = 0.027, s.e. = 0.014, *t*-value = 1.938, *p* = 0.052), its interaction with temperature was significant (*β* = −0.025, s.e. = 0.009, *t*-value = −2.744, *p* = 0.006; electronic supplementary material, figure S1). Visitation time was shorter for sequence 1 (4.5 ± 9.0 s) and sequence 2 (4.2 ± 9.8 s) in 32°C than for sequence 1 (7.9 ± 14.5 s; *β* = 0.088 and *β* = 0.09, s.e. = 0.006 and 0.014, *z* = 14.202 and 6.318, *p* < 0.001 for both), sequence 2 (6.3 ± 12.0 s; *β* = 0.061 and *β* = 0.063, s.e. = 0.015 and 0.007, *z* = 4.17 and 9.55, *p* < 0.001 for both) and sequence 3 (7.0 ± 13.8 s; *β* = 0.071 and *β* = 0.073, s.e. = 0.015 and 0.014, *z* = 4.69 and 5.28, *p* < 0.001 for both) in 24°C.

### (c) Flight speed

**Figure 4.**
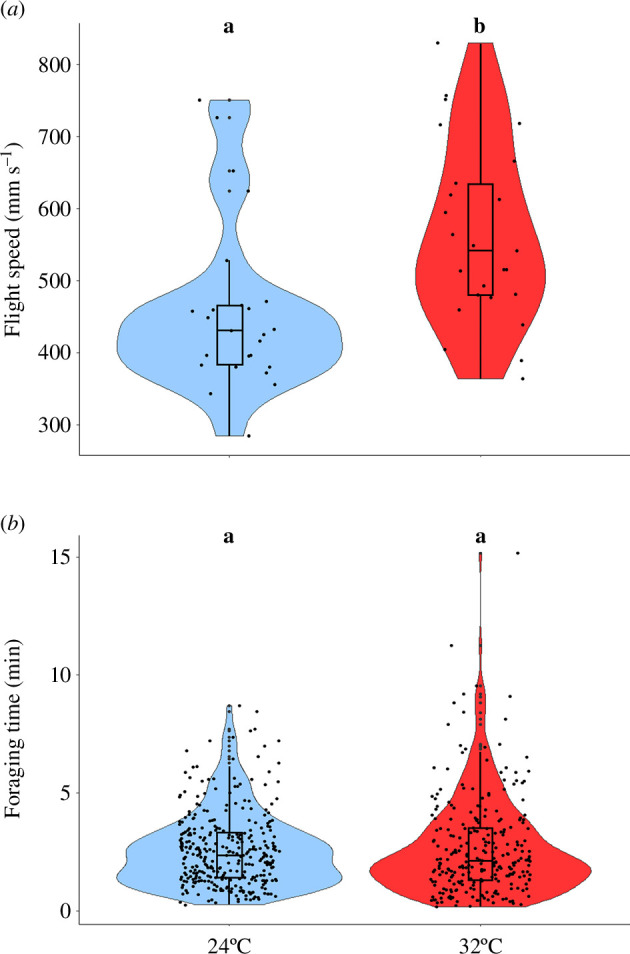
Impact of ambient temperature on flight speed (*a*; *β* = 79.76, s.e. = 22.51, *t*-value = 3.54, *p* < 0.001) and foraging time (*b*; *β* = 0.05, s.e. = 0.06, *t*-value = 0.89, *p* = 0.375), results based on four colonies (A to D). Each dot represents data from one individual. Letters above the boxplots indicate significant differences when the letters are different. The central line within the boxplots represents the median. The upper and the lower lines represent the lower (Q1) and the upper (Q3) quartiles, respectively. The whiskers extend from Q1 and Q3 up to 1.5 times the interquartile range.

We recorded flight speed from a total of 51 flights from 48 individuals. The model that best explained variation in speed included temperature as a fixed factor and colony ID as a random factor (next best model ΔAIC 2.12; electronic supplementary material, table S3). Workers flew significantly faster at 32°C (551 ± 81 mm s^−1^) than at 24°C (472 ± 88 mm s^−1^; *β* = 79.76, s.e. = 22.51, *t*-value = 3.54, *p* < 0.001; figure 4*a*).

### (d) Foraging time

Foraging time was recorded for 596 flights from 105 different individuals. The model explaining log foraging time included temperature treatment as a fixed factor and experimental block and individual ID as random factors (next best model ΔAIC 0.1; electronic supplementary material, table S4). Foraging time was not affected by temperature (*β* = 0.05, s.e. = 0.06, *t*-value = 0.89, *p* = 0.375; 24°C: 155 ± 95 s; 32℃: 162 ± 126 s; figure 4*b*).

### (e) Foraging trips

The number of trips made by each individual per trial was counted, with a total of 767 values being included in the analysis (from 134 different individuals). The model that best explained the number of foraging trips included temperature as a fixed factor and block, colony and individual ID nested in the colony as random factors (next best model ΔAIC 1.72; electronic supplementary material, table S5). There was some indication that temperature affected the number of foraging trips performed per individual (*β* = 0.097, s.e. = 0.057, *t*-value = 1.696, *p* = 0.089), with fewer trips recorded at 32°C than at 24°C (1.7 ± 1.3 versus 1.9 ± 1.3) (electronic supplementary material, figure S2).

### (f) Treatment level data

At the treatment level, fewer foraging trips were recorded at 32°C than at 24°C (594 versus 820; electronic supplementary material, figure S3A) but more workers were performing these trips (109 versus 94; electronic supplementary material, figure S3B), indicating that, across the experiments, each worker performed fewer foraging trips at 32°C than at 24°C (5.45 versus 8.72).

### (g) Flight metabolic rate

**Figure 5.**
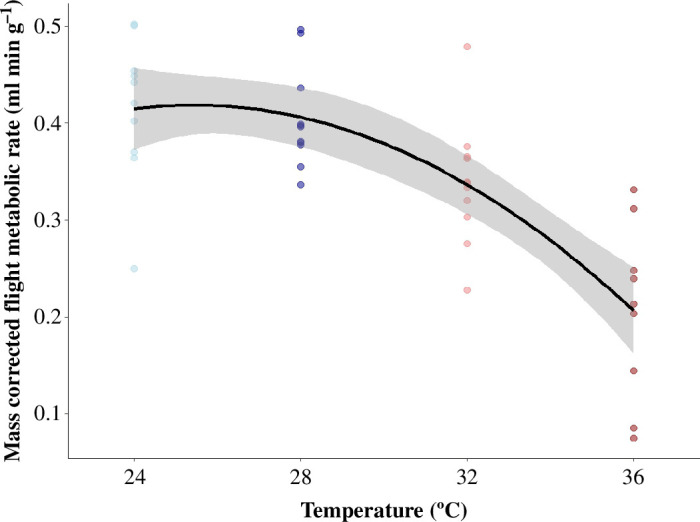
Flight metabolic rate of bumblebee workers across temperatures (*β* = −0.096, s.e. = 0.013, *t*-value = −7.451, *p* < 0.001). Colours correspond to increasing environmental temperatures (blue to red), *n* = 10 at 24°C, *n* = 11 at 28°C, *n* = 12 at 32°C and *n* = 9 at 36°C. Grey shaded area around the line indicates the 95% CI.

We recorded the flight metabolic rates of 42 individuals across a range of temperatures from 24°C to 36°C. Metabolic rate was best explained by a model that included the quadratic polynomial of temperature treatment, colony ID and their interaction and body mass as fixed factors (next best model ΔAIC 1.9; electronic supplementary material, table S6). Our analysis indicated that flight metabolic rate decreased significantly with temperature (*β* = −0.096, s.e. = 0.013, *t*-value = −7.451, *p* < 0.001; figure 5).

## 4. Discussion

By maintaining colonies at a constant, optimal temperature and by using artificial flowers, we directly investigated the effects of ambient temperature on bumblebee foraging behaviour. Our results indicate that less than 3 min (i.e. the average time of one foraging trip in our set-up) exposure to elevated ambient temperature is sufficient to impact several important aspects of foraging behaviour in bumblebees and that this effect remains after repeated exposures (i.e. when two 32°C treatments were intermingled with one 24°C treatment). These changes in behaviour are consistent with the reduction in flight metabolic rate we recorded but also indicate that flight in elevated temperatures does come at the cost of heat stress.

The higher visitation rate that we observed at 32°C is likely due to the significant increase in flight speed and decrease in visitation time recorded at this temperature. When subjecting both plants and solitary bees to elevated temperatures, de Manincor *et al.* [14] observed a decrease in visitation rate. However, in this experiment, the plants suffered heat stress and produced fewer flowers, which could have increased the time required for the bees to find flowers and handle them. In our study, the effect of temperature on plants was not an issue as the food rewards were standardized. It is nonetheless important to highlight that, in the wild, both plants and pollinators will be affected by changes in temperature, although more detailed studies on these interactions are necessary to determine exactly if or how plant–pollinator relationships might be affected. For example, workers made more flower visits per unit time at 32°C but, with real plants, such an increase in visitation rate might not necessarily translate to an increase in pollination efficiency [28], which depends on the quantity and quality of pollen deposits [38]. Additionally, we did not measure the evaporation rate of the sucrose solution. Higher temperatures cause sucrose solutions to evaporate more quickly, resulting in more concentrated solutions that slow down bumblebee feeding rates, meaning they take longer to consume the same volume [39]. This decrease in feeding rate would theoretically increase visitation time, which contradicts our observations. Therefore, we conclude that potential differences in the evaporation rate of the sucrose solution at the two temperatures, over the 30 min experimental session, do not explain the observed changes in foraging behaviour. A limitation to this set-up could be that, even if bees did not completely empty a flower during the 30 min trial, an artificial flower might be less attractive if it contains a smaller volume of nectar. In various pollinator species, the number of visits increases when the nectar volume is higher [40,41]. A more standardized approach to our experiment would thus be to use automatic artificial flowers that consistently display the same volume of nectar (e.g. [42]).

Our finding that flight speed and visitation rate increased at 32°C compared to 24°C, while foraging time remained constant, could be explained by the reduction in flight metabolic rate we recorded with increasing temperatures. Bumblebees need to heat up their flight muscles to 30–40°C before flying [43]. Thus, the energy expenditure needed to reach this thoracic temperature at 32°C would be less than at 24°C. We hypothesize that, as a result, bumblebees were able to fly faster and visit more flowers at 32°C without expending more energy than they did at 24°C. It has previously been shown that the metabolic rate of bumblebees remains constant from hovering to forward speeds of 4.5 m s^−1^, suggesting that faster flight does not come at an increased energetic cost [44,45]. Considering this, energetic costs may not have limited flight speed in our experiment but the extra energetic costs associated with an increased visitation rate could potentially be limited by energy, as each take-off would require additional power output. In addition, if the bees take a constant amount of nectar during each visit, then each take-off would require increasingly more power, and therefore energetic cost, due to the increase in body weight. As we did not measure the amount of sugar water consumed during each visit, we cannot determine if foragers in the 32°C condition returned home with more nectar. Exploring the relationship between ambient temperature and the amount of nectar brought back to the colony by individual foragers would make for an interesting focus in future studies.

Interestingly, while the number of workers that made foraging trips from each colony increased with temperature, each worker performed fewer trips. Remarkably similar effects of temperature on foraging behaviour were observed in Gérard *et al.* [46]. In that previous study, however, the foraging behaviour was recorded at the same ambient temperature (25°C) but the foragers had experienced either optimal (27°C) or elevated temperatures (33°C) during their development from pupa to adult. Workers that developed under an elevated temperature of 33°C made fewer foraging trips and the number of foraging trips per worker decreased in comparison to those that developed at an optimal temperature (27°C). Whether experienced in the colony during development or outside it while foraging, these findings suggest that exposure to elevated temperature constrains the number of trips each worker is capable of making and that this is likely due to the effects of heat stress. As nearly the same amount of heat is generated during flight at varying temperatures [47], a higher speed could be a way to increase convective cooling [48] without any energetic cost. However, additional experiments are necessary to tease apart the different causalities. It is possible that the colony as a whole reacted to the variations in ambient temperature (despite being maintained at a constant and optimal temperature in both treatments), something that could potentially be driven by factors relating to the increased body temperatures of the returning foragers. For example, Sepúlveda-Rodríguez *et al.* [49] recently showed that the internal temperatures of worker *B. terrestris* are elevated above ambient temperature after flight at 32°C. Thus, the foragers flying at 32°C could have been returning to the colony with a significant ‘thermal baggage’ that could potentially be perceived and responded to by workers inside the colony, leading to a colony-level thermal response. This hypothesis still needs further investigation. Overall, our findings are consistent with those of Hemberger *et al.* [17], who found that *B. impatiens* workers had a reduced proportion of foraging trips relative to the number of attempts made to leave the colony at 35°C. The authors argue that this could be due to perceived potential heat stress, which could cause workers to spend longer inside the colony and increase worker turnover, as bumblebees have been shown to make heat avoidance trade-offs [50].

With fewer trips per worker, more workers are required to forage, which would then limit the number of workers available to perform other duties in the colony. Bumblebees, like other flying pollinators, need to both choose flowers and handle them efficiently to avoid a net loss of resources [51,52]. Therefore, fewer foraging trips performed by a larger number of workers could result in slower learning of best flower choices and handling techniques to gather resources efficiently [53], which could affect colony growth and pollination services. In addition, exposure to elevated temperature can impair memory and learning in *B. terrestris* [15], which could accentuate the negative effects of slower learning. These two factors could have a combined impact on bumblebee foraging in the wild where the environment is much more complex and foraging trips could be performed for a much longer duration [54] than in our set-up. The flowers in this study were all identical in traits and rewards (although the sugar reward of some flowers may have decreased during a trial if many workers fed on them), meaning that any choice of flower would be equally rewarding and require the same effort. If flower traits had differed, like in nature, it is possible that successful localization and extraction of nectar could have been affected by ambient temperatures due to an impairment of cognition and reduced experience.

This study was conducted in a laboratory setting with commercial colonies and, although it was designed to allow natural foraging behaviour, many parameters were unlike those that would be experienced in nature (e.g. lighting, space, flower traits). Under natural conditions, other factors would likely play a role in either buffering against or enhancing the impacts of temperature. Elevated ambient temperatures will not only impact bumblebees but also the flowering plants that they feed on, colony conditions and other biological interactions (e.g. [55,56]). Elevated temperatures can result in smaller corollas and reduced nectar production in flowers, leading to fourfold decrease in flower visits [57]. While we observed an increase in visitation rates with artificial flowers where morphology is constant, this trend could be completely reversed if heat also impacts the plant itself. This could negatively affect pollination and reduce the quantity of resources brought back to the colony. Additionally, elevated temperatures are often accompanied by reduced water availability. Höfer *et al.* [58] highlighted that short-term drought stress could alter bumblebee behaviour and interact with temperature, which could exacerbate the effects we observed. Combined heat and drought can significantly alter floral morphology and rewards, particularly causing a marked decrease in nectar and pollen volume [59]. To more accurately assess the impact of changes in pollinator visitation rates on plant fitness, future studies should focus on measuring pollination efficiency using real flowers. In addition, under field conditions, ambient temperature fluctuates, unlike in our study where it was stable. There are no studies comparing the impact of fluctuating versus stable temperatures on the foraging behaviour of pollinators. This area deserves more attention, as several studies on other insect species have shown contrasting results regarding fitness (e.g. [60,61]). Further, *B. terrestris* is a species with a Mediterranean-centred distribution in Europe [62]. Due to their partial endothermy, bumblebees are well adapted to cool environments and therefore mostly confined to temperate, arctic and alpine habitats. Seeing the impact that experiencing an elevated temperature of 32℃ during two discontinuous weeks had on the foraging activity of *B. terrestris* raises concern as to how bumblebee species that are less adapted to warm climates (e.g. Arctic species) will be affected by rising temperatures and highlights the need for broader comparative studies on this topic. Finally, we also emphasize the importance of studying the synergy between stressors. Kenna *et al.* [63] recently demonstrated that while some stressors alone may have no detectable effects on behaviour, their combination (e.g. pesticides and temperature) could have a much more pronounced impact. We thus encourage researchers to explore the effects of heat on pollinator behaviour more thoroughly, as there are still many unexplored aspects of this topic. The increasing impact of global warming underscores the urgency of addressing these gaps in our knowledge.

## Data Availability

The R code and dataset related to this paper are available on Dryad [64]. Supplementary material is available online [65].
